# Changes in TNF and IL-6 production after diphtheria toxoid vaccination: drug modulation of the cytokine levels

**DOI:** 10.1155/S0962935196000592

**Published:** 1996-12

**Authors:** A. L. Pukhalsky, M. S. Bliacher, A. V. Danilina, E. A. Kalashnikova, T. K. Lopatina, I. M. Fedorova

**Affiliations:** 1 Research Centre for Medical Genetics 1 Moskvorechie Street Moscow 115478 Russia; 2 Moscow Institute of Epidemiology and Microbiology Moscow Russia; 3 National Institute of Epidemiology Moscow Russia

## Abstract

The effect of vaccination with diphtheria toxoid (AD-M) on TNF and IL-6 production has been studied in humans. In the present study it was demonstrated that immunization with AD-M resulted in changes of *in vitro* TNF and IL-6 production by peripheral blood mononuclear cells. TNF release was suppressed but IL-6 production was stimulated. On the other hand, serum levels of TNF were markedly increased over a period of 3 weeks. It was also demonstrated that the postvaccinal cytokine production disturbances may be corrected by pretreatment with a new synthetic hexapeptid (Imunofan^®^). It is possible that the imunofan treatment could prevent some postvaccinal complications.


Mediators of Inflammation, 5, 429–433 (1996)

### Changes in TNF and IL-6 production after diphtheria toxoid vaccination: drug modulation of the cytokine levels

A. L. Pukhalsky,[1,CA] M. S. Bliacher,[2] A. V. Danilina,[3] E. A. Kalashnikova,[1] T. K. Lopatina[2] and I. M. Fedorova[2]

[1]Research Centre for Medical Genetics, 1 Moskvorechie Street, 115478, Moscow; [2]Moscow Institute of Epidemiology and Microbiology; [3]National Institute of Epidemiology, Moscow, Russia

[CA]Corresponding Author
Fax: (+7) 095 324 0702

**THE effect of vaccination with diphtheria toxoid (AD-M) on TNF and IL-6 production has been studied in humans. In the present study it was demonstrated that immunization with AD-M resulted in changes of *in vitro* TNF and IL-6 production by peripheral blood mononuclear cells. TNF release was suppressed but IL-6 production was stimulated. On the other hand, serum levels of TNF were markedly increased over a period of 3 weeks. It was also demonstrated that the post-vaccinal cytokine production disturbances may be corrected by pretreatment with a new synthetic hexapeptid (Imunofan®). It is possible that the imunofan treatment could prevent some post-vaccinal complications.**

**Key words:** Diphtheria toxoid, Imunofan, Interleukin-6, Tumour necrosis factor, Vaccination

### Introduction

The safety of vaccination in the first instance depends on the vaccine's characteristics. It is well known, however, that the vaccine's application always switches on a cascade of events which results in cytokine release. Thus, the spontaneous production of high levels of IL-4 and TNF-α after measles virus vaccination has been shown.[1] The transient increase of IL-6 in serum has been also demonstrated after vaccination with brucella antigenic extracts and live, attenuated *Francisella tularensis* in mice and humans.[2,3] Murine spleen cells taken at intervals after infection and cultured with brucella antigens produced elevated levels of IL-1, IL-6 and TNF-α[2]. These cytokines play an important role in the development of inflammation.[4] The inflammatory reactions, which are safe for healthy individuals, may entail serious consequences in children with a variety of forms of immune disturbance. Production of at least one of the aforesaid proinflammatory cytokines may be regulated with a new immunomodulating agent thymohexin (Imunofan®). This synthetic hexapeptid is a modified analogue of the thymopoietin II active centre.[5] The inhibitory effect of imunofan (IF) on TNF-α production in septic patients has been shown.[6]

In the present study, we investigated TNF and IL-6 production after diphtheria toxoid vaccination and the possibility of cytokine level modulation by IF pretreatment.

### Material and Methods

### Subjects

Seventeen healthy adult volunteers (male and female; mean age 30.4 years, range 22–54 years) were selected on the basis of at least a 10-year period without revaccination.

### Vaccine

Absorbed diphtheria toxoid with low content (10 LF/ml) of antigens (AD-M) was obtained from BIOMED (Petrovo-Dal'neie, Russia).

### Immunomodulating hexapeptid

Imunofan (arginyl-α-aspartyl-lysyl-valyl-tyrosyl-arginine) synthetic modified analogue of thymopoietin II active centre was obtained from Bionox-Bios (Moscow, Russia).

### Vaccination

All volunteers were divided into three groups. The volunteers of the first group (five subjects) received 0.5 ml AD-M; the individuals of the second and third group (six subjects in each)

© 1996 Rapid Science Publishers received AD-M and 1 ml (0.05 mg) IF or IF only. Both AD-M and IF were mixed in the same syringe and injected subcutaneously within the shoulder-blade region.

### Blood collection

Blood was collected in heparin (25 IU/ml) and in dry tubes (for serum collection) before immunization and 1, 7, 14, 30 and 120 days after immunization.

### Cells and cultures

Peripheral blood mononuclear cells (PBMNCs) were isolated from heparinized peripheral blood by Ficoll-Verographin gradient sedimentation. The cells were washed twice and resuspended in RPMI-1640 medium (ICN, UK) supplemented with 10% heat inactivated donor horse serum, $2 \times 10^{-3}$ M HEPES, 2 mM L-glutamine, $2.8 \times 10^{-6}$ M 2-mercaptoethanol, and 20 $\mu$g/ml gentamycin. Cells ($10^6$ cells/ml) were cultivated for 2 or 14 h at 37°C in a humidified atmosphere containing 5% $CO_2$ in the wells (1.5 ml per well) of 24-well plates (Nunc, Denmark). The supernatants were collected and stored at −20°C until cytokine activity examination.

### TNF activity assay

TNF activity was determined by the method of Ruff and Gifford[7] with some modifications. Briefly, L929 cells were seeded at a density $3 \times 10^4$ cells per well in 96-well plates in 100 µl of medium 199 to which 10% heat inactivated calf bovine serum and gentamycin had been added. Plates were incubated at 37°C in a humidified atmosphere containing 5% $CO_2$ until the monolayer formation. After the culture medium elimination, two-fold serial dilution of the samples (100 µl of each dilution) and 100 µl fresh culture medium with 2 µg/ml of actinomycin D (Serva, Germany) were added, and further incubated for 18 h at the same conditions. Supernatants were then removed and cells stained with 0.2% crystal violet (Sigma, USA). After washing and drying plates were finally read at 540 nm on a Titertek Multiskan micro-Elisa reader. Human recombinant TNF (Institute of Bioorganic Chemistry, Moscow, Russia) was used as internal standard. For the comparison of an experimental and calibrating curves probit-analysis method was used. TNF content in the samples was expressed in pg/ml.

### IL-6 activity assay

IL-6 activity was determined using IL-6-dependent hybridoma cell line D6C8.[8] Briefly, serial dilutions of culture supernatants and recombinant IL-6 (code 89/45, NIBSC, UK) as a standard, were incubated in 96-well microplates with cells ($5 \times 10^4$ cells/well), in a total volume of 200 µl at 37°C. The cells were cultivated for 48 h in RPMI-1640 medium supplemented with 5% human dialysed AB-serum. Four hours before the end of cultivation the cells were pulsed with 40 kBq per well of [$^3$H]-thymidine, harvested with a cell harvester and counted by using a liquid scintillation counter.

### Antitoxic antibody assay

Indirect haemagglutination with diphtheria toxoid attached to erythrocytes has been performed. Antitoxic antibody titre were determined using a commercial kit obtained from BIOMED (Petrovo-Dal'neie, Russia).

### Statistical analysis

Statistical comparison were performed using the Wilcoxon–Mann–Whitney's $U$ criterion, Student's $t$-test, and Fisher's exact test.

### Results

### Changes in TNF production

As a rule LPS treatment stimulated TNF production compared with untreated cultures but in some cases such stimulation was not observed. There was also donor-to-donor variability in the levels of cytokine production before vaccination (Table 1). In view of this fact our experimental data was presented as a percentage of control.

AD-M (with or without IF) strongly suppressed spontaneous and LPS-induced TNF production by PBMNCs at the 7th day after injections. The suppression was maintained at the 14th day in subjects injected with AD-M only, but those who received the mixture of AD-M and IF demonstrated TNF production restoration until the initial level (Fig. 1). Individuals of the third group (IF injection only) showed a significant LPS-induced TNF production increase ($p < 0.025$ in Fisher's exact test). The increase of spontaneous TNF production was also observed in volunteers of the second group (AD-M + IF) 4 weeks after vaccination ($p < 0.025$ in Fisher's exact test).

Serum levels of TNF were markedly increased in four out of five individuals who received

**Table 1.** TNF and IL-6 production levels before vaccination

| Group no. | Subject no. | TNF (pg/ml) | | IL-6 (IU/ml) | |
|---|---|---|---|---|---|
| | | Without LPS | With LPS | Without LPS | With LPS |
| I | 1 | 67 | 80 | 956 | 1687 |
| (AD-M) | 2 | 305 | 230 | 1242 | 1357 |
| | 3 | 1017 | 1185 | 1012 | 800 |
| | 4 | 817 | 3734 | 1197 | 1707 |
| | 5 | 1187 | 1320 | 1020 | 750 |
| II | 6 | 131 | 591 | 1621 | 4073 |
| (AD-M + IF) | 7 | 136 | 587 | 1455 | 2971 |
| | 8 | 15 | 20 | 465 | 1405 |
| | 9 | 301 | 1529 | 1362 | 2138 |
| | 10 | 30 | 62 | 922 | 2169 |
| | 11 | 24 | 83 | 173 | 261 |
| III | 12 | 139 | 687 | 1327 | 2645 |
| (IF) | 13 | 780 | 1972 | 1072 | 2130 |
| | 14 | 661 | 1128 | 380 | 1337 |
| | 15 | 458 | 990 | 853 | 515 |
| | 16 | 157 | 317 | 243 | 320 |
| | 17 | 76 | 166 | 807 | 473 |

AD-M only as a comparison with initial levels. No changes in serum levels of TNF have been demonstrated in both AD-M with IF and IF only injected subjects (Fig. 2).

### Changes in IL-6 production

The IL-6 production levels before vaccination are shown in Table 1. Evident stimulation of IL-6 production (both spontaneous and LPS-induced) at the 14th day after AD-M application has been observed. No significant differences with an initial IL-6 production were obtained for individuals injected with mixture of AD-M and IF or with IF only (Fig. 3).

In general, the serum level of IL-6 in vaccinated volunteers receiving IF was lower than in individuals injected with AD-M only (Fig. 4).

### Antitoxic antibody titre

High antitoxic antibody initial titres were shown in five volunteers. Serum antibody levels lower than the protective titre (1:40) have been demonstrated in the others. The results indicate that systemic antibody responses to diphtheria toxoid vaccination were similar in both groups of volunteers, with and without IF pretreatment. However, the dynamics of antibody formation were different in the groups (Fig. 5). Thus, in the control group the velocity of antibody accumulation and maximum antibody titres in the sera were higher than in the group pretreated with IF. But 120 days after immunization serum antigen-specific antibody titres in IF-pretreated individuals did not differ from those in the controls.

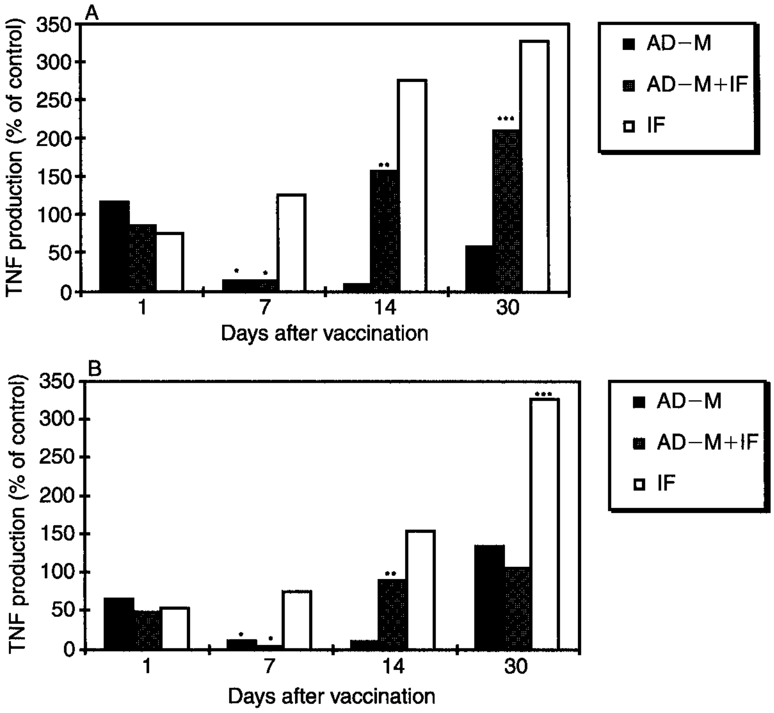

FIG. 1. The effect of AD-M, AD-M + IF, and IF only on *in vitro* TNF production by PBMNCs. PBMNCs obtained from donors of different groups were incubated for 2 h without (panel A) or with (panel B) LPS. The supernatants were collected and TNF activity was quantified as described in Materials and Methods. *$p < 0.05$ compared with control value; **$p = 0.021$ compared with suppressed TNF level at the 7th day (Wilcoxon–Mann–Whitney's $U$ criterion). ***The use of Fisher's exact test led us to reject the hypothesis of random stimulation of TNF production ($p < 0.025$).

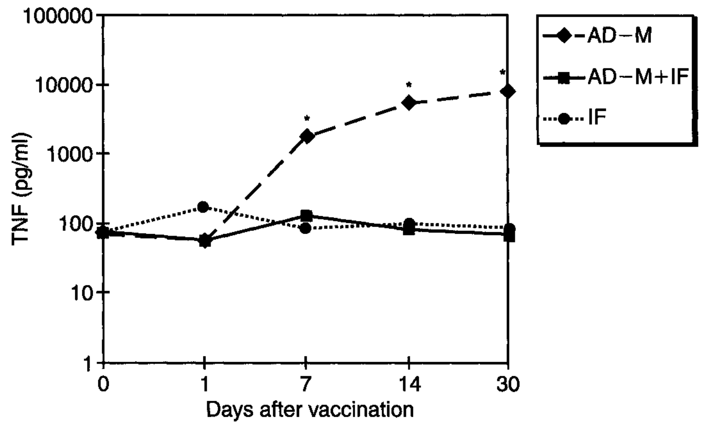

FIG. 2. The effect of AD-M, AD-M + IF, and IF only on serum TNF levels. The average values of TNF levels in the sera of different individuals are presented. $*p < 0.05$ compared with respective control (Wilcoxon–Mann–Whitney's *U* criterion).

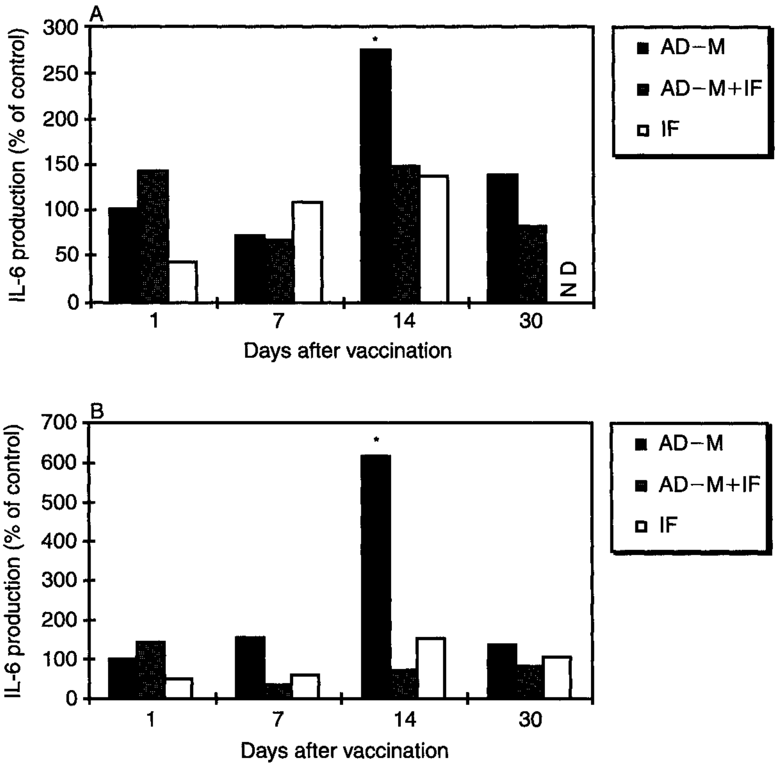

FIG. 3. The effect of AD-M, AD-M + IF, and IF only on *in vitro* IL-6 production by PBMNCs. PBMNCs obtained from donors of different groups were incubated for 14 h without (panel A) or with (panel B) LPS. The supernatants were collected and IL-6 activity was quantified as described in Materials and Methods. $*p < 0.05$ compared with control value (Wilcoxon–Mann–Whitney's *U* criterion).

### Discussion

Our data show that visible changes in the proinflammatory cytokine system are detectable after a single application of diphtheria toxoid in low dose. The vaccination resulted in changes of TNF and IL-6 production by PBMNCs. Thus, TNF release was suppressed and IL-6 production was stimulated. Similar antagonistic production of TNF to production of IL-6 has been observed in the sera of patients with acute cerebral ischaemia[9] and during the course of meningococcal infections.[10] It is well known that TNF and IL-6 play different roles in the immune response mechanisms. It was shown, for example, that IL-6 can induce the production of TNF and IL-1 antagonists.[11]

Despite the TNF production being suppressed, serum levels of the cytokine were markedly increased over a period of 3 weeks after vaccination. This contradiction may be

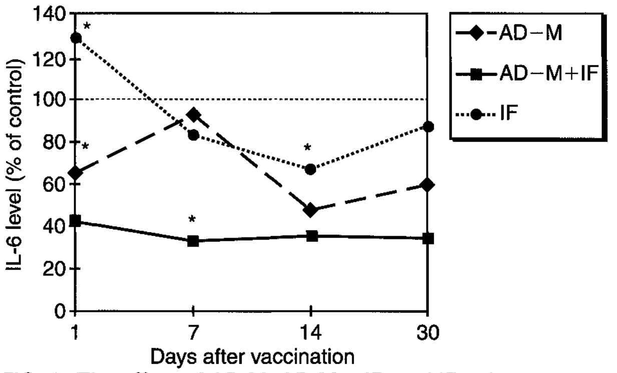

FIG. 4. The effect of AD-M, AD-M + IF, and IF only on serum IL-6 levels. The average values of IL-6 levels in the sera of different individuals are presented. $*p < 0.05$ compared with respective control (Wilcoxon–Mann–Whitney's *U* criterion).

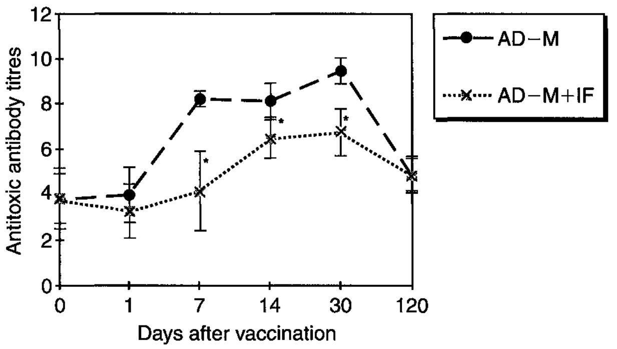

FIG. 5. Time course of serum antitoxic antibody responses. $*p < 0.05$ *vs* antibody titres after AD-M application without IF (Student's *t*-test).

explained by some peculiarities of our experimental model. With the supernatant levels of TNF measured 2 h after PBMNC isolation, only the cytokine release from cellular depots has been observed. In view of this fact the high levels of serum TNF may correlate with cellular depot depletion.

Our results also demonstrate that the pharmacological correction of postvaccinal cytokine production disturbances is quite possible. Both the stimulation of the suppressed TNF production and inhibition of the elevated IL-6 release have been shown. Normalization of the serum TNF level in IF-treated and vaccinated subjects has been also demonstrated. It appears that IF can act in a dualistic manner on inflammatory cytokine production, the elevated production is suppressed and the low one is stimulated. A similar effect of IF has been shown in septic patients.[6] On the other hand, a single injection of IF delays the antigen-specific antibody growth, although the protective titres of antitoxic antibodies has been revealed in all vaccinated subjects.

In prospect these data may be useful for the prevention of postvaccinal complications in children with neuro- and/or immunopathology which can demonstrate inadequate response to elevated levels of proinflammatory cytokines in the blood.

### References

1. Ward BJ, Griffin DE. Changes in cytokine production after measles virus vaccination: predominant production of IL-4 suggests induction of Th2 response. *Clin Immunol Immunopathol* 1993; **67**: 171–177.
2. Zhan Y, Kelso A, Cheers C. Cytokine production in the murine response to brucella infection or immunization with antigenic extracts. *Immunology* 1993; **80**: 458–464.
3. Krakauer T. Levels of interleukin 6 and tumor necrosis factor in serum from humans vaccinated with live, attenuated *Francisella tularensis*. *Clin Diagn Lab Immunol* 1995; **2**: 487–488.
4. Cavaillon JM. La participation des cytokines au cours des mecanisms inflammatoires. *Pathol Biol Paris* 1993; **41**: Part 2, 799–811.
5. Lebedev VV, Ivanushkin EF, Maksimov SL, Pokrovsky VI. Introduction of new drug containing synthetic peptide for clinical evaluation in the patients with chronic hepatitis B. In: *Symposium Franco-Soviétique de la Biotechnologie*. Moscow: Institute of Bioorganic Chemistry, 1990; 142.
6. Pisarev V, Leoshin A, Tutelian A, Danilina A, Kremlev S, Lebedev V, Semenov V. Septic inflammation and control of TNF spontaneous production. In: *12th European Immunology Meeting*. Barcelona: 1994; 126.
7. Ruff MR, Gifford GE. Tumor necrosis factor. In: Pick E, ed. *Lymphokines*. New York: Academic Press. 1981; 235–241.
8. Kliushenkova EN, Malaitzev VV. Establishment of an interleukin-6(IL-6)-dependent hybridoma. *Bull Exp Biol Med* 1992; **114**: 179–181.
9. Fassbender K, Rossol S, Kammer T, Daffertshofer M, Wirth S, Dollman M, Hennerici M. Proinflammatory cytokines in serum of patients with acute cerebral ischemia: kinetics of secretion and relation to the extent of brain damage and outcome of disease. *J Neurol Sci* 1994; **122**: 135–139.
10. van Deuren M, van der Ven Jongerkrijg J, Demacker PN, Bartelink AK, van Dalen R, Sauerwein RW, Gallati H, Vannice JL, van der Meer JW. Differential expression of proinflammatory cytokines and their inhibitors during the course of meningococcal infections. *J Infect Dis* 1994; **169**: 157–161.
11. Tilg H, Trehu E, Atkins MB, Dinarello CA, Mier JW. Interleukin-6 (IL-6) as an anti-inflammatory cytokine: induction of circulating IL-1 receptor antagonist and soluble tumor necrosis factor receptor p55. *Blood* 1994; **83**: 113–118.

ACKNOWLEDGEMENT. The authors thank I. Kapustin for expert technical assistance.

**Received 9 August 1996; accepted in revised form 30 September 1996**

## References

[PDF_00408] Cavaillon J. M. (1993). La participation des cytokines au cours des mécanismes inflammatoires.. Pathol Biol (Paris).

[PDF_00423] Fassbender K., Rossol S., Kammer T., Daffertshofer M., Wirth S., Dollman M., Hennerici M. (1994). Proinflammatory cytokines in serum of patients with acute cerebral ischemia: kinetics of secretion and relation to the extent of brain damage and outcome of disease.. J Neurol Sci.

[PDF_00405] Krakauer T. (1995). Levels of interleukin 6 and tumor necrosis factor in serum from humans vaccinated with live, attenuated Francisella tularensis.. Clin Diagn Lab Immunol.

[PDF_00433] Tilg H., Trehu E., Atkins M. B., Dinarello C. A., Mier J. W. (1994). Interleukin-6 (IL-6) as an anti-inflammatory cytokine: induction of circulating IL-1 receptor antagonist and soluble tumor necrosis factor receptor p55.. Blood.

[PDF_00399] Ward B. J., Griffin D. E. (1993). Changes in cytokine production after measles virus vaccination: predominant production of IL-4 suggests induction of a Th2 response.. Clin Immunol Immunopathol.

[PDF_00402] Zhan Y., Kelso A., Cheers C. (1993). Cytokine production in the murine response to brucella infection or immunization with antigenic extracts.. Immunology.

[PDF_00428] van Deuren M., van der Ven-Jongekrijg J., Demacker P. N., Bartelink A. K., van Dalen R., Sauerwein R. W., Gallati H., Vannice J. L., van der Meer J. W. (1994). Differential expression of proinflammatory cytokines and their inhibitors during the course of meningococcal infections.. J Infect Dis.

